# Return to sport and patient reported outcomes in athletes participating in martial arts after anterior cruciate ligament reconstruction at mean follow‐up of 12 years

**DOI:** 10.1002/jeo2.70272

**Published:** 2025-05-19

**Authors:** Yunseo Linda Park, Anja M. Wackerle, Brooke Collins, Ehab M. Nazzal, Joseph D. Giusto, Matthew Kolevar, James J. Irrgang, Jonathan D. Hughes, Volker Musahl

**Affiliations:** ^1^ University of Pittsburgh School of Medicine Pittsburgh Pennsylvania USA; ^2^ UPMC Freddie Fu Sports Medicine Center Pittsburgh Pennsylvania USA; ^3^ Department for Orthopaedic Sports Medicine, Klinikum Rechts der Isar Technical University of Munich Munich Germany; ^4^ UPMC Department of Orthopaedic Surgery Pittsburgh Pennsylvania USA; ^5^ Department of Physical Therapy University of Pittsburgh Pittsburgh Pennsylvania USA

**Keywords:** anterior cruciate ligament, anterior cruciate ligament reconstruction, martial arts, martial arts athletes, outcomes, return‐to‐sport

## Abstract

**Purpose:**

This study aims to assess anterior cruciate ligament reconstruction (ACLR) outcomes and return to pre‐injury sports (RTPS) characteristics in athletes participating in martial arts.

**Methods:**

Martial arts athletes over the age of 14 years who underwent primary ACLR with a minimum 1‐year follow‐up were eligible for this study. This study defined RTPS as reaching pre‐injury levels of martial arts participation. Patients completed a questionnaire assessing sports participation, reinjury, and patient reported outcomes (PROs) including International Knee Documentation Committee Subjective Knee Form (IKDC SKF), Marx activity score, Tegner activity scale, and visual analogue scale (VAS). Demographics, surgical data, and preoperative PROs were collected retrospectively. Patients were grouped into those who achieved RTPS and those who did not. Statistical analyses included chi‐square, Fisher's exact, Mann–Whitney *U*, and t‐tests. Statistical significance was set at *p* < 0.05.

**Results:**

Fifty‐two individuals who participated in martial arts (mean age 30.6 ± 11.0 years, 21% female) completed the questionnaire at a mean follow‐up of 12.1 ± 7.8 years. Of the cohort, 35 patients (67%) participated in competitive, varsity, or elite martial arts prior to injury. Of those who did not return to martial arts at all, fear of reinjury was the most common reason. The rate of RTPS was 58%. Age, pre‐injury martial arts participation (frequency and competitiveness), and surgical characteristics did not differ between groups. Those who did not achieve RTPS had a higher rate of minor and major postoperative complication (41% vs. 13%, *p* = 0.02), which included stiffness, infection, effusion, and reinjury. The RTPS group reported statistically higher IKDC SKF scores (71.4 ± 11.7 vs. 68.4 ± 7.2, *p* < 0.01) and Tegner activity scores (6.9 vs. 5.6, *p* = 0.04) at final follow‐up. Graft failure rate for all study participants was 12% and did not differ between groups (*p* = 0.69). Reinjury rate of the RTPS group was 17%.

**Conclusion:**

Martial arts athletes demonstrated a rate of 58% return to pre‐injury participation levels after ACLR.

**Level of evidence:**

Case series; level IV.

**Abbreviations**
ACL: anterior cruciate ligament
ACLR: anterior cruciate ligament reconstruction
ACL‐RSI: ACL return to sport after injury
IKDC SKF: International Knee Documentation Committee Subjective Knee Form
MMA: mixed martial arts
PRO: patient reported outcomes
RTPS: return to pre‐injury sports
RTS: return to sport
VAS: visual analogue scale

## INTRODUCTION

Anterior cruciate ligament (ACL) injuries are one of the most prevalent injuries in active sports and can have a profound impact on athletes' careers and long‐term knee health. Returning to sport and being able to reach pre‐injury level of sports participation has been recognised as a key outcome for athletes following their initial injury [4]. Success and timing of return to pre‐injury sports (RTPS) after ACL reconstruction (ACLR) depend on many factors. For instance, sports that require significant pivoting and cutting manoeuvres place substantial stress on the knee joint, often leading to lower RTPS rates [14]. Secondary injuries that cause ACL graft failure or contralateral ACL tears are common adverse outcomes that can prevent full RTPS. Active athletes have a greater propensity for a secondary ACL injury [18], and this increased risk is further pronounced within the first 2 years of return to sport (RTS) [2]. ACL injuries can carry significant physical and psychological impacts on athletes and can ultimately jeopardise athletic careers if not managed properly.

Martial arts consist of a variety of combat sports and are characterised by full‐contact sparring. The demands of martial arts include quick pivoting and high‐impact manoeuvres, which can predispose athletes to ACL injuries. ACL injuries are among the most common injuries that lead to significant time lost from participation in martial arts, with higher level of experience and competition associated with a higher injury incidence [27]. In judo, for example, ACL injuries were found to be predominantly contact injuries, often occurring during direct attacks by opponents. This suggests that the nature of the sport, which involves frequent grappling and sudden directional changes, contributes to the high incidence of ACL injuries [1, 27]. Additionally, due to these unique physical requirements, martial arts may pose specific challenges for following ACLR and post‐operative rehabilitation and RTS performance. A recent study on 48 mixed martial arts (MMA) fighters showed significantly declined defensive performance upon RTS when compared to a matched cohort, representing the impact of ACLR on an athlete's performance level despite RTS [8]. While outcomes and factors influencing RTS after ACLR have been extensively studied in athletes of other contact sports, research on RTS of athletes participating in martial arts remains limited.

The purpose of this study was to identify the rate of RTPS for athletes participating in martial arts following ACLR. For this study, RTPS is operationally defined as the return to pre‐injury level of performance in martial arts. Additionally, this study sought to elucidate reasons athletes were unable to RTPS as well as investigate the relationship between patient reported outcomes (PROs) and RTPS. This study hypothesised that > 50% of athletes will achieve successful RTPS and those who return to their pre‐injury level of sport will report higher IKDC scores, lower complication rates, and reduced psychological barriers to sport re‐engagement than those who do not reach their pre‐injury level of sport.

## METHODS

This retrospective study was approved by the institutional review board. All patients who underwent primary ACLR between 1987 and 2023 by 1 of 7 fellowship‐trained orthopaedic sports medicine surgeons were reviewed. Inclusion criteria consisted of patients greater than 14 years of age with a first time ACL injury who self‐reported they participated in one or more of the following types of martial arts prior to first ACL injury: wrestling, MMA, karate, taekwondo, judo, kung fu, jujitsu, Muay Thai, aikido, boxing or kickboxing. Exclusion criteria included age <14 years at surgery, follow‐up length <1 year, prior ipsilateral knee surgery, ≥2 concomitant ligamentous injuries requiring operative intervention, concomitant osteotomy, lateral extra‐articular tenodesis (LET), or meniscus allograft transplantation.

ACLR was recommended for young, active individuals who wished to return to martial arts and high‐demand activities that involve pivoting, cutting, or jumping. The choice of graft type in ACLR was influenced by patient's age, activity level, and specific needs. All patients underwent arthroscopic ACLR with an anatomic technique [7]. A 30° arthroscope was used for diagnostic arthroscopy to assess the knee and determine the need for additional procedures such as meniscectomy or meniscus repair, as deemed necessary by the surgeon. The native ACL femoral and tibial footprints were directly visualised before drilling bone tunnels, which were positioned within these footprints. Fixation methods for both femoral and tibial sites were at the surgeon's discretion and included various combinations of suspensory and interference screw fixation. The knee flexion angle and graft tension during fixation varied among surgeons but were typically set near full knee extension. Patients followed a scheduled postoperative follow‐up plan and were initially referred to the institution's rehabilitation facilities. Postoperative rehabilitation protocols, including bracing, were determined by the surgeon but generally followed a structured progression of range of motion, strength, and functional training over 9–12 months.

All eligible patients were contacted via email and phone call; written informed consent was acquired upon enrolment of the patient. Enroled participants were asked to complete a questionnaire on their demographic information including age, sex, body mass index, martial arts type, level and frequency of martial arts participation before and after injury, injury data, return to martial arts, return to work and postoperative complications (Supporting Information: S1). Patients who sustained an ACL graft failure, defined as revision ACLR, during the study period and/or underwent >1 ACLR were asked to base their responses on their recovery from their primary ACLR. Patients were also asked to complete the following patient‐reported outcome measures > International Knee Documentation Committee Subjective Knee Form (IKDC SKF), Marx Activity Acore, Tegner Activity Scale, and visual analogue scale (VAS) pain scores. Questionnaires were distributed and completed using an institutional REDCap database (REDCap, Vanderbilt University). Follow‐up length was determined by the date of questionnaire completion.

Surgical characteristics including graft type and concomitant meniscal procedures were collected via chart review. Additional outcomes collected retrospectively included rates of subsequent revision or contralateral ACLR and surgical characteristics such as graft type and concomitant meniscal procedures.

Patients were stratified into two groups depending on whether they successfully RTPS, defined as returning to pre‐injury levels or higher. Demographics, surgical characteristics, post‐operative outcomes, and PROs were compared between groups.

### Statistical methods

All analyses were conducted using SPSS software version 28.0.1.1 (IBM). Descriptive statistics were reported for all demographic variables, postoperative outcomes, and survey elements, and displayed as means with standard deviations or numbers with percentages. Comparisons between groups were assessed using the chi‐square and Fisher's exact tests for categorical variables, or Mann–Whitney *U* tests and independent sample t‐tests for nonparametric and parametric variables, respectively. Significance level was set at *p *< 0.05.

## RESULTS

**Figure 1:**
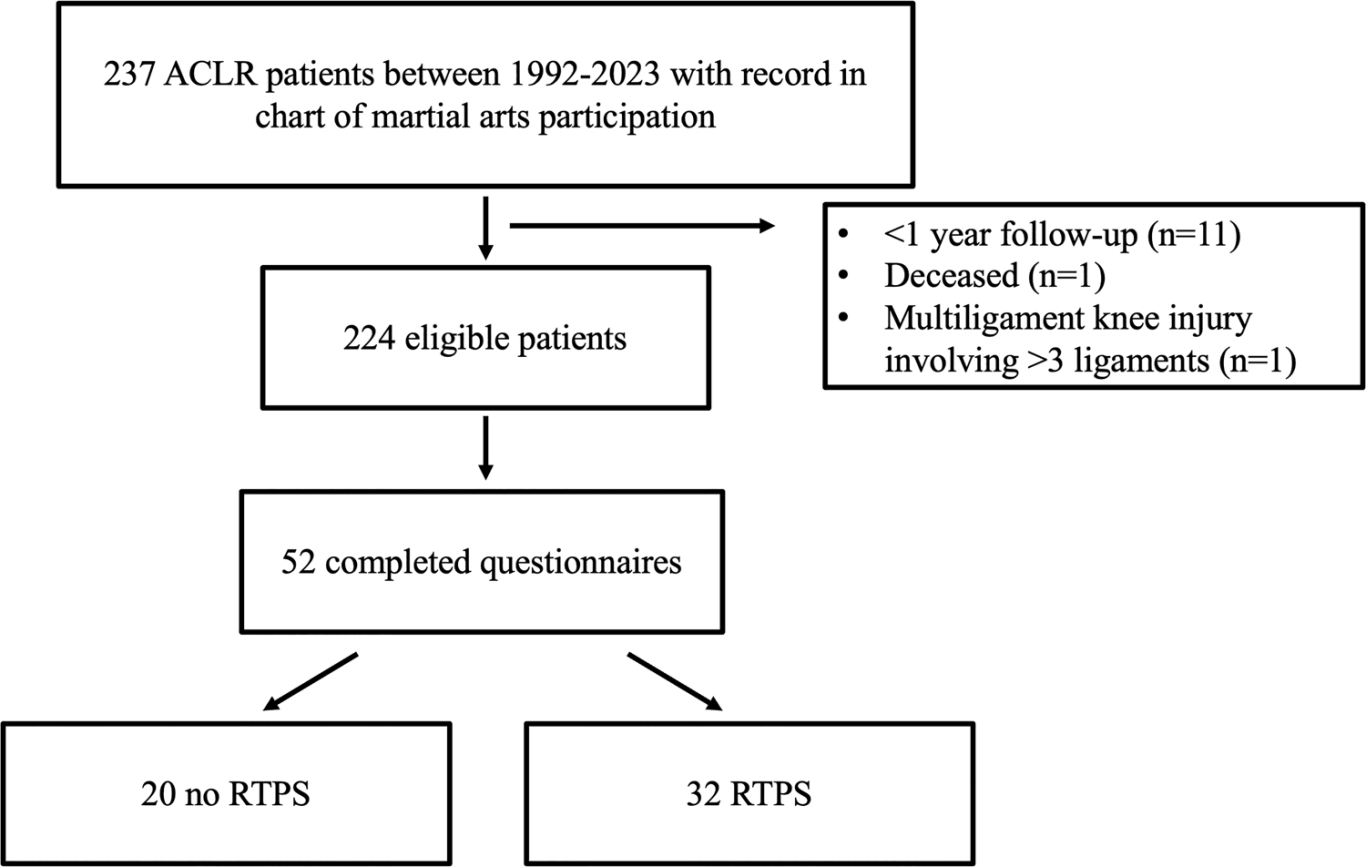
Flowchart of patient inclusion. ACLR, anterior cruciate ligament reconstruction; RTPS, return to preinjury sports.

A total of 237 primary ACLR patients with a record of martial arts participation in their chart were identified. Eleven patients were excluded due to < 1 year follow‐up, one was deceased, and one had a multiligament injury involving three ligaments. The remaining 224 patients who met inclusion criteria were contacted, of which 52 patients (23%) completed questionnaires (Figure 1). This cohort had a mean age at surgery of 30.6 ± 11.0 years (range: 12.3–53.5, median (IQR): 31.0 (20.0–38.9)) and mean follow‐up length of 12.1 ± 7.8 years (range: 1.1–37.1, median (IQR): 10.9 (6.7–15.9)). Of note, 35 patients (67%) were competitive, varsity, or elite martial arts athletes at pre‐injury based on survey data.

### Overall cohort

Of the entire cohort, 30 patients (58%) reached their pre‐injury levels or higher. Twelve patients (23%) did not return to martial arts at all. Reasons cited for not returning to martial arts included non‐ACL related reasons for five patients (42%), fear of reinjury for four patients (33%), persistent subjective knee instability for two patients (17%), and knee pain for one patient (8%). The 40 patients (77%) who returned to any level of martial arts answered additional questions regarding the time it took to return to training and competition, which can be found in Table 1. Of the 40 who returned to some level of martial arts, 22 (55%) participated in competition after ACLR. A total of 27 patients reported a pre‐injury belt level (11 black, 2 red, 1 brown, 2 purple, 4 blue, 3 green, 1 orange and 3 white); eight patients (30%) obtained a higher belt after ACLR, 11 (41%) maintained the same belt level after ACLR, 7 (26%) did not RTS, and 1 (4%) did not report a post‐injury belt level. In terms of return to work, 47 patients (90%) were able to return to work to pre‐injury levels. The remainder of return‐to‐work data can be found in Table 2. Surgical outcomes of the overall cohort are reported in Table 3. Ten patients (20%) had a second ACL surgery, either due to ACL graft failure or ACL injury in the contralateral knee. Specifically, ACL graft failure rate was 12%, and the contralateral ACL injury rate was 8%.

**Table 1:** Time to return to martial arts training and competition after ACLR.

	*n* (%) (*n* = 40)^a^
Light training (non‐contact, no sparring, drills only)	
Within 3 months	4 (10)
Within 6 months	13 (33)
Within 9 months	13 (33)
Within 12 months	6 (15)
More than 12 months	4 (10)
Heavy training (sparring, contact included)	
Within 3 months	1 (3)
Within 6 months	7 (18)
Within 9 months	14 (35)
Within 12 months	10 (25)
More than 12 months	8 (20)
Competition level	
Within 12 months	13 (33)
Within 1.5 years	6 (15)
Within 2 years	2 (5)
More than 2 years	1 (3)
No participation in competition prior to ACL injury	4 (10)
No return to competition level due to ACL‐injury related reasons	5 (13)
No return to competition level due to non‐ACL reasons	9 (23)

Abbreviations: ACL, anterior cruciate ligament; ACLR, anterior cruciate ligament reconstruction.

a Characteristics are reported among survey respondents who returned to martial arts regardless of reaching pre‐injury levels.

**Table 2:** Return to work characteristics of entire cohort.

	*n* (%)
Return to work to same level, *n* (%)	47 (90)
Pre‐injury, *n* (%)	
Employment status	
Regular duty	37 (71)
Light duty or modified position	1 (2)
Not currently working	14 (27)
Work type and lift requirements	
Very heavy	4 (8)
Heavy	2 (4)
Moderately heavy	6 (12)
Medium	8 (15)
Light	18 (35)
Post‐injury, *n* (%)	
Employment status	
Regular duty	43 (83)
Not currently working	8 (15)
Permanently unable to work due to health status	1 (2)
Work type and lift requirements	
Very heavy	8 (15)
Heavy	2 (4)
Moderately heavy	2 (4)
Medium	8 (15)
Light	23 (44)

*Note*: Characteristics are reported among all survey respondents regardless of returning to sports.

**Table 3:** Surgical outcomes of entire cohort.

	*n* (%)
Post‐operative characteristics	
Complication^a^	13 (25)
Length of physical therapy	
Less than 3 months	15 (29)
3–6 months	14 (27)
6–9 months	11 (21)
9–12 months	5 (10)
Greater than 12 months	7 (14)
Subsequent injury	
Any knee injury, ipsilateral	9 (17)
ACL injury, ipsilateral	10 (19)
ACL injury, contralateral	4 (8)
Subsequent ACL surgery	
Ipsilateral knee	6 (12)
Contralateral knee	4 (8)

*Note*: Characteristics are reported among all survey respondents regardless of returning to sports.

Abbreviation: ACL, anterior cruciate ligament.

a Complications described further in text.

### RTPS versus no RTPS cohort

There were 30 individuals that achieved the designation of RTPS group and 22 individuals that did not achieve RTPS. All demographic factors, comorbidities, and surgical characteristics were similar between groups (Table 4).

**Table 4:** Baseline characteristics of patients who did and did not return to preinjury level of participation.

	No RTPS (*n* = 22)	RTPS (*n* = 30)	*p*‐Value
Demographics			
Age at time of ACLR (years), median (IQR)	21.4 (18.5–40.4)	26.0 (21.3–41.8)	0.41
Female sex	7 (32)	4 (13)	0.17
Laterality right	11 (50)	13 (45)	0.71
BMI (kg/m^2^) at time of ACLR (*n* = 34), median (IQR)	27.4 (22.1–33.5)	25.2 (22.3–29.0)	0.96
Race			
White	21 (96)	28 (93)	0.82
Black or African American	0 (0)	1 (3)	
Other	1 (5)	1 (3)	
Ethnicity			
Non‐Hispanic/Latino	19 (86)	26 (87)	1.00
Hispanic/Latino	1 (5)	1 (3)	
Chose not to disclose	2 (9)	3 (10)	
Follow‐up in years, median (IQR)	7.9 (2.0‐12.4)	8.5 (5.5‐10.4)	0.71
Time between injury and surgery in months (*n* = 44), median (IQR)	3.1 (0.9‐4.0)	1.8 (1.3‐4.4)	0.77
Comorbidities			
No comorbidities	13 (59)	22 (73)	0.28
Diabetes	0 (0)	3 (10)	0.25
Hypertension	6 (27)	6 (20)	0.54
High cholesterol	3 (14)	2 (7)	0.64
Osteoarthritis	4 (18)	1 (3)	0.15
Kidney disease	0 (0)	0 (0)	‐
Cardiovascular disease	0 (0)	0 (0)	‐
Current smoker or vaper	3 (14)	2 (7)	0.64
History of smoking or vaping	0 (0)	4 (13)	0.13
Surgical characteristics (*n* = 47)			
Graft type			
Bone patellar bone tendon autograft	3 (14)	5 (19)	0.47
Quadriceps autograft	7 (33)	9 (35)	
Hamstring autograft	3 (14)	7 (27)	
Allograft	8 (38)	5 (19)	
Concomitant meniscus surgery	6 (32)	12 (46)	0.59
Any meniscus repair	2 (10)	6 (23)	0.62
Lateral meniscus repair	1 (5)	2 (8)	1.00
Medial meniscus repair	1 (5)	4 (15)	0.64
Any meniscectomy	4 (19)	8 (31)	0.85
Lateral meniscectomy	2 (10)	7 (27)	0.46
Medial meniscectomy	2 (10)	2 (8)	1.00
Concomitant ligament injury	4 (21)	3 (12)	0.43
MCL	4 (21)	3 (12)	0.43
PCL	1 (5)	0 (0)	0.68
Concomitant ligament surgery	3 (16)	1 (4)	0.30
MCL	3 (16)	1 (4)	0.30

*Note*: Data reported for entire cohort unless noted by *n* = number of patients with available data. Data presented as number (%) unless otherwise indicated.

Abbreviations: ACLR, anterior cruciate ligament reconstruction; BMI, body mass index; IQR, interquartile range (25th–75th percentile); PRO, patient reported outcome; VAS, Visual Analogue Scale.

### Return to martial arts participation and injury mechanism

Wrestling was the leading type of martial arts that individuals participated in both groups (No RTPS: 46%, RTPS: 50%), followed by Taekwondo in the No RTPS group (32%) and mixed martial arts (20%) and karate (20%) in the RTPS group. Many individuals participated in more than one type of martial arts. Of note, more patients in the RTPS group participated in karate compared to those that were not able to RTPS (20% vs. 0%, *p *= 0.03). Characteristics of martial arts participation prior to ACL injury were similar between groups (Table 5). Specifically, level of competitiveness of martial arts participation prior to injury was similar between groups, with 14 (64%) in the No RTPS group and 21 (70%) in the RTPS group participating at a competitive, varsity, or elite level. While more patients in the RTPS group had a belt level blue or above, this difference was not statistically significant (No RTPS: 27%, RTPS 47%; *p* = 0.33). Regarding injury mechanism, 14 (82%) of the No RTPS group and 19 (79%) of the RTPS group had a contact martial arts injury. When these patients were asked about the fighting manoeuvre that caused the injury, seven patients (21%) noted that they were being taken down, five patients (15%) were taking their opponent down, one patient (3%) was passing guard, three patients (9%) were sweeping their opponent, four patients (12%) were being swept, and 12 patients (36%) described their injury in free text. Some of these descriptions included landing from a jump, throwing a kick, pivoting while on one leg, and swinging their legs out of the ring.

**Table 5:** Martial arts participation and ACL injury mechanism.

	No RTPS (*n* = 22)	RTPS (*n* = 30)	*p*‐Value
Martial arts type, *n* (%)			
Wrestling	10 (46)	15 (50)	0.75
Mixed martial arts (MMA)	3 (14)	6 (20)	0.72
Karate	0 (0)	6 (20)	**0.03** ^a^
Taekwondo	7 (32)	5 (17)	0.20
Judo	2 (9)	4 (13)	1.00
Kung Fu	1 (5)	2 (7)	1.00
Jujitsu	1 (5)	3 (10)	0.63
Brazilian jujitsu	2 (9)	7 (23)	0.27
Muay Thai	2 (9)	2 (7)	1.00
Aikido	0 (0)	3 (10)	0.25
Boxing	3 (14)	3 (10)	0.69
Kickboxing	2 (9)	4 (13)	1.00
Other	3 (14)	4 (13)	1.00
Pre‐injury martial arts participation			
Level of sport, organised (competitive, varsity or elite)	14 (64)	21 (70)	0.63
Elite	1 (5)	5 (17)	0.34
Varsity	8 (36)	12 (40)	
Competitive	5 (23)	4 (13)	
Recreational	6 (27)	9 (30)	
Nonorganized	2 (9)	0 (0)	
Belt level, blue or above (*n* = 27)	6 (27)	14 (47)	0.33
Frequency of participation			
4–7 times per week	16 (73)	20 (67)	0.88
1–3 times per week	5 (23)	9 (30)	
1–3 times per month	1 (5)	1 (3)	
Considers martial arts as primary sport	18 (82)	24 (80)	1.00
Years of experience before injury, median (IQR)	9.0 (3.0‐12.3)	7.5 (2.0‐12.0)	0.94
ACL injury			
Injured during martial arts^a^	17 (77)	24 (80)	1.00
Contact martial arts injury (*n* = 41)	14 (82)	19 (79)	1.00
Martial arts ACL injury setting (*n* = 41)			
Warm‐up	1 (6)	1 (4)	0.76
Drilling technique	2 (12)	5 (21)	
Sparring	9 (53)	9 (38)	
Competition	5 (29)	9 (38)	
Post‐injury martial arts participation (*n* = 40)			
Frequency of participation			
4–7 times per week	1 (8)	7 (25)	0.65
1–3 times per week	3 (25)	5 (18)	
1–3 times per month	1 (8)	4 (14)	
Less than 1 time per month	7 (58)	12 (43)	
Martial arts participation affected by knee			
Not at all	2 (17)	18 (64)	**0.02** ^a^
Mildly	5 (42)	6 (21)	
Moderately	3 (25)	2 (7)	
Severely	1 (8)	0 (0)	
Extremely	1 (8)	2 (7)	

*Note*: Data reported for entire cohort unless noted by *n* = number of patients with available data or applicable for that variable. Data presented as number (%) unless otherwise indicated.

Abbreviations: ACL, anterior cruciate ligament; IQR, interquartile range (25th–75th percentile).

a Martial arts injury mechanism described further in text.

### Postoperative outcomes

Following ACLR, the group that was unable to RTPS had more complications than the group who did RTPS (41% vs. 13%, *p* = 0.02). Complications in the RTPS group included chondrosis, knee stiffness, effusion and pulmonary embolism, while complications in the No RTPS group included femoral nerve injury, patellar maltracking, knee stiffness, graft failure prior to RTS attempt (*n* = 2), infection, effusion, and readmission due to compartment syndrome. Distribution of the length of postoperative physical therapy differed between groups; more patients in the RTPS group had physical therapy for less than 3 months postoperatively (40% vs. 14%, *p* = 0.02). In comparison to those that were not able to RTPS, those that RTPS had statistically significantly greater PRO values for IKDC SKF (*p* < 0.01), Tegner activity scale (*p* = 0.04), and change in Tegner from baseline to most recent follow‐up (*p* = 0.03). The mean IKDC SKF scores were 68.4 and 71.4 for the RTPS and no RPTS groups, respectively. Both groups had scores lower than the normative values for an age‐matched population, which is 84.9 in males age 35–50 and 79.9 in females age 35–50 [3, 23]. There were no statistically significant differences in VAS (*p* = 0.59) and Marx (*p* = 0.10) scores between groups (Table 6). When asked to what degree their knee injury still affected their participation in martial arts (not at all, mildly, moderately, severely, or extremely), the distribution of responses differed significantly between groups (*p* = 0.02) (Table 5). Occurrences of subsequent knee injuries and revision ACLR were not significantly different between groups (Table 6). Specifically, graft failure rates were 13% and 9% in the RTPS and no RTPS groups, respectively (*p* = 0.69).

**Table 6:** Return to sport characteristics.

	No RTPS (*n* = 22)	RTPS (*n* = 30)	*p*‐Value
Post‐operative characteristics			
Complication^a^	9 (41)	4 (13)	**0.02** ^a^
Length of physical therapy			
Less than 3 months	3 (14)	12 (40)	**0.02** ^a^
3–6 months	6 (27)	8 (27)	
6–9 months	3 (14)	8 (27)	
9–12 months	4 (18)	1 (3)	
Greater than 12 months	6 (27)	1 (3)	
Subsequent injury			
Any knee injury, ipsilateral	4 (19)	5 (17)	0.32
ACL injury, ipsilateral	5 (23)	5 (17)	
ACL injury, contralateral	3 (14)	1 (3)	
Subsequent ACL surgery			
Ipsilateral knee	2 (9)	4 (13)	0.69
Contralateral knee	3 (14)	1 (3)	0.30
PROs at most recent follow‐up, median (IQR)			
IKDC SKF total	70.7 (64.4–72.7)	74.7 (71.0–77.0)	**<0.01** ^a^
VAS	0 (0–2.3)	0 (0–1.0)	0.59
Tegner activity scale (*n* = 50)	6.0 (4.3–7.0)	7.0 (5.0–9.0)	**0.04** ^a^
Change in Tegner from pre‐injury to final follow‐up (*n* = 50)	−2.0 (−3.8 to −1.0)	0 (−3.0 to 0)	**0.03** ^a^
Marx	7.5 (0–12.0)	10.0 (4.0–14.0)	0.10
Global rating of change			
Better	12 (55)	14 (47)	0.69
About the same	6 (27)	12 (40)	
Worse	4 (18)	4 (13)	

*Note*: Data reported for entire cohort unless noted by *n* = number of patients with available data. Data presented as number (%) unless otherwise indicated.

Abbreviations: ACL, anterior cruciate ligament; PRO, patient reported outcome; SD, standard deviation; VAS, Visual Analogue Scale.

a Postoperative complications described further in text.

## DISCUSSION

The main finding of the present study was that 58% returned to their pre‐injury levels (RTPS), and 42% returned to competition. The 58% who RTPS reported lower adverse outcome rates and statistically significantly greater PROs than those who did not return to pre‐injury levels. The overall rate of a second ACL injury in either knee was 20%, but this did not differ between those who were, vs. those who were not able to successfully return to pre‐injury sports participation.

A recent systematic review that included nine studies of collegiate athletes reported an RTS rate that ranged from 69% to 92%, with a cumulative RTS rate of 84% [6]. Another cohort study of 222 patients who injured their ACL while participating in a sport reported that 61% returned to pre‐injury levels of performance after ACLR [29]. However, both studies included participants from various sports and no sport‐specific analyses were performed. Sport‐specific studies are crucial, as different sports place unique physical demands on the knee and require distinct movement patterns, training intensities and biomechanical loads. A 2024 study reviewed RTS in soccer players, finding that 72% returned to play and 53% returned to pre‐injury levels [16]. Mean RTS time was 8.7 months, and performance generally decreased compared to pre‐injury. A cohort study on National Football League (NFL) defensive players demonstrated an RTS rate of 74% to at least one game and 61% to at least half a season [24]. A systematic review of high‐level athletes and RTPS ability showed that there are measurable decreases in performance statistics that are highly sport‐specific [20]. Another systematic review on RTS after ACLR included 24 studies, 18 of which reported on a cohort of elite athletes from a single sport, but none of those were martial arts [11]. This review reported a pooled RTS rate of 83%, with a mean time to RTS 6–13 months. A recent study of MMA fighters with ACLR reported that 81% returned for at least one fight, and 56% returned for at least two fights after ACLR [8]. The present study differs, as it includes PROs at 12 years after surgery as well as clinical outcomes such as postoperative complications and secondary injuries.

The RTPS rates reported by this study are also similar to studies evaluating martial arts injuries other than the ACL. For general orthopaedic injuries, approximately 60% of martial arts athletes return to their pre‐injury level of participation [12, 13, 22]. A case series of 20 martial arts athletes with anterior shoulder instability reported that 60% achieved ≥ 90% recovery after arthroscopic stabilisation with suture anchors. Another case series of 50 athletes participating in judo who underwent open capsular shift reported that 65% of their cohort recovered to at least 90% of pre‐injury activity levels in judo [28].

This study highlights that sustaining a postoperative adverse outcome can significantly affect one's ability to RTPS. Types of adverse outcomes experienced by the current study's cohort varied widely. Adverse outcomes in the No RTPS group seemed to be more severe than those in the RTPS group, such as graft failure in the rehabilitation period, infection, nerve injury, stiffness requiring debridement or lysis, and readmission due to compartment syndrome. These complications may have contributed to an inability to RTPS or heightened fear of returning. In contrast, adverse outcomes in the RTPS group included effusion, stiffness requiring debridement or lysis, and pulmonary embolism.

In addition to postoperative adverse outcomes, secondary ACL surgery, either due to graft failure or contralateral ACL injury, was a common outcome in this cohort, affecting 20% of athletes. The self‐reported rate of secondary ACL injury regardless of surgical intervention in either knee was 27%—slightly above rates reported in the literature. A systematic review of secondary ACL injuries showed an overall rate of 15%, which increased to 20% among those who returned to sport [30]. Possible reasons for the higher rate in the present study may include the extended follow‐up period or the unique movements of the sport such as single‐leg stability challenges and hyperextension from high kicks and blocks. However, these remain hypotheses, and further research is needed to clarify why secondary ACL injuries might be more frequent in martial arts athletes.

In order to address the need to control rotational instability of the knee, surgeons have begun performing LETs in addition to ACLRs. A recent study that aimed to identify factors influencing RTS and graft failure rates in professional football players after ACLR demonstrated that the lowest graft rupture rate was observed in patients with patella tendon autografts in an anteromedial bundle femoral tunnel position with the addition of LET [5]. Additionally, studies that compared athletes with and without an LET procedure showed that combining ACLR with LET can lead to a significantly lower risk of graft rupture and need for reinterventions [9, 10, 26].

Another key finding of this study was that athletes who achieved RTPS reported significantly higher IKDC scores than those who did not RTPS. However, both groups had IKDC scores below population norms. A recent cross‐sectional, international, multicenter study established normative values for Knee injury and Osteoarthritis Outcome (KOOS) and Western Ontario and McMaster Universities Osteoarthritis Index (WOMAC) scores [17]. Similarly, another cross‐sectional survey of 5246 knees from a representative sample of the US population determined normative values of IKDC scores [3], which was used as a reference point for interpreting scores of the present study.

The PROs used in this study (IKDC SKF, Marx, Tegner, VAS) focus primarily on the physical and functional aspects of the individual's experience, which may overlook the psychological impact of the knee injury on the athlete. Fear of reinjury emerged as the major concern for athletes to return to martial arts in this cohort of individuals participating in martial arts, and previous studies have emphasised that psychological readiness is associated with return to sport [25, 31] as well as a secondary ACL injury [19]. Martial arts are unique in having a “combat” nature in the context of sparring and competition, which may contribute as another barrier to psychological readiness. This underscores the need for comprehensive rehabilitation protocols that address both the physical and psychological aspects to optimise RTS outcomes. For instance, athletes may benefit from additional psychological counselling before clearance for RTS. Interestingly, age, competition level, and time to surgery did not differ between groups, unlike other studies that have identified these factors as predictors of RTPS [21].

This study had several limitations. First, the low response rate increased the risk of nonresponse bias and selection bias, as patients who participated in the survey may differ from those who did not in terms of motivation, function, or injury severity. Second, despite the benefit of long‐term follow‐up > 10 years post‐injury, this does come with an inherent risk of recall bias. Third, psychological aspects were not assessed via a validated questionnaire such as the ACL Return to Sport after Injury (ACL‐RSI) survey [15], which may be utilised in future studies to help evaluate the role of psychological readiness in RTS for the martial arts athlete. Finally, while some PROs were statistically significantly different between groups, their clinical significance is unknown as both groups had scores lower than the normative scores of an age‐matched population.

## CONCLUSION

Athletes participating in martial arts demonstrated favourable long‐term clinical outcomes after ACLR, with 58% achieving pre‐injury participation levels. Those who achieved RTPS reported less complications and significantly better functional outcomes. Findings of this study are useful for surgeons treating this unique patient population and can aid in counselling and managing expectations for RTS in both the short and long‐term setting.

## AUTHOR CONTRIBUTIONS

All authors contributed to the study conception and design. Material preparation, data collection and analysis were performed by Yunseo Linda Park, Anja M. Wackerle, and Brooke Collins. The first draft of the manuscript was written by Yunseo Linda Park, and all authors commented on previous versions of the manuscript. All authors read and approved the final manuscript.

## CONFLICT OF INTEREST STATEMENT

V.M. received consulting fees from Smith & Nephew and Newclip, stock or stock options from Ostesys, and is a board member of the International Society of Arthroscopy, Knee Surgery and Orthopaedic Sports Medicine (ISAKOS) and a Deputy Editor of Knee Surgery, Sports Traumatology, Arthroscopy (KSSTA). V.M. is the 1st Vice President for the ACL Study Group. V.M. has grant funding from the NIH and DOD, but this is not pertinent to this current study. V.M. has a patent, U.S. Patent No. 9,949,684, issued on 24 April 2018. J.D.H. is on the editorial board of Knee Surgery, Sports Traumatology, Arthroscopy. J.D.H. has received grant support from Arthrex; education payments from Mid‐Atlantic Surgical Systems, Smith & Nephew, and Medical Device Business Services; and hospitality payments from SI‐Bone. J.J.I. is President of the Board of Directors for the Journal of Orthopaedic and Sports Physical Therapy.

## ETHICS STATEMENT

The questionnaire and methodology for this study was approved by the University of Pittsburgh Institutional Review Board (STUDY23080187). Informed consent was obtained from all individual participants included in the study.

## Supporting information

Supplementary Information 1.

## Data Availability

The data that support the findings of this study are available from the corresponding author upon reasonable request.
