# Supplementary material for: Return to sport and patient reported outcomes in athletes participating in martial arts after anterior cruciate ligament reconstruction at mean follow‐up of 12 years
Source: J Exp Orthop. 2025 May 19;12(2):e70272. doi: 10.1002/jeo2.70272 (PMC12086811; doi:10.1002/jeo2.70272)
Supplement: Supplementary file 1 — Supplementary Information 1. [file JEO2-12-e70272-s001.pdf]

Supplementary Information 1. Full version of questionnaire completed by included patients

Demographics

Please complete the survey below.Thank you!

Please answer the following question(s). If a "End the Survey?" message pops up, please click the "End Survey" button.

Did you participate in martial arts or combat sports leading up to your ACL injury? (Includes wrestling, boxing, jujitsu, taekwondo, judo, taekwondo, etc.)

If you stopped participating in martial arts months or years before your ACL injury occurred OR started martial arts after your ACL reconstruction, please answer no.

- ◯ Yes
- ◯ No
- ◯ Other

If other, please explain.

____

Who was your surgeon for your ACL reconstruction? (If multiple ACL surgeries, please record ALL surgeons' last names to the best of your ability.)

____

How many ACL reconstructions did you have TOTAL? (Include revision ACL reconstructions due to re-rupture/re-injury.)

- ◯ 1
- ◯ 2
- ◯ 3
- ◯ 4
- ◯ 5 or more

Are you open to receiving a phone call or text messages for additional questions regarding your survey responses, in case of need for clarification, etc.?

- ◯ Yes
- ◯ No

Preferred phone number

____

Email address

____

Demographics

Sex assigned at birth:

- ◯ Male
- ◯ Female

How do you identify?

- ◯ Man
- ◯ Non-binary
- ◯ Woman
- ◯ Prefer to self-describe, below

Self-describe:

____

Ethnicity:

◯ Hispanic or Latino
◯ Not Hispanic or Latino
◯ Unknown or Unavailable

Race:

◯ American Indian or Alaska Native
◯ Asian
◯ Black or African American
◯ White
◯ Native Hawaiian or Other Pacific Islander
◯ Other
◯ Unknown or Unavailable

Marital Status:

◯ Married
◯ Single
◯ Divorced
◯ Separated
◯ Widowed
◯ Unknown or Unavailable
(At the time of surgery)

Employed:

◯ Yes
◯ No
(At the time of surgery)

Occupation:

____
(At the time of surgery)

Health Information

Height:______

Inches

Weight:______

lbs

Have you ever been diagnosed with any of the following conditions?

☐ Diabetes
☐ High Blood Pressure
☐ High Cholesterol
☐ Osteoarthritis
☐ Kidney Disease
☐ Cardiovascular Disease (Heart attack, stroke, heart failure)
☐ None
(Select all that apply)

Do you currently smoke cigarettes or vape?

☐ I currently smoke cigarettes
☐ I currently vape
☐ I no longer smoke
☐ I no longer vape
☐ I have never smoked
☐ I have never vaped

How many years did you smoke or vape for?

____

How many years AND months have passed since you last smoked or vaped?

____
(Y/M)

On average, how many cigarettes did you smoke per day?

- ◯ 1-10 (less than half a pack)
- ◯ 11-20 (half a pack to one pack)
- ◯ 21-40 (one to two packs)
- ◯ More than 40 (two packs)
- ◯ I only vaped

Sports participation PRIOR to ACL injury

What type(s) of martial arts did you participate in PRIOR to your ACL injury?

- ☐ Wrestling
- ☐ Mixed martial arts (MMA)
- ☐ Karate
- ☐ Taekwondo
- ☐ Judo
- ☐ Kung Fu
- ☐ Jujitsu
- ☐ Brazilian Jiu-jitsu
- ☐ Muay Thai
- ☐ Aikido
- ☐ Boxing
- ☐ Kickboxing
- ☐ Other

If other, please describe.

____

What belt did you have in this sport PRIOR to your ACL injury?

____

How many years of experience did you have prior to your injury?

____

At what level did you play this sport?

- ◯ Elite: Playing at the highest level of professional competition
- ◯ Varsity: Playing for a team representing a U.S. High School, College, or University
- ◯ Competitive: Playing for a competitive league
- ◯ Recreational: Playing for a beginner or social league
- ◯ Non-Organized: Not playing for a league; playing casually with friends or family

How often did you participate in this sport or activity?

- ◯ 4 to 7 times per week
- ◯ 1 to 3 times per week
- ◯ 1 to 3 times per month
- ◯ Less than one time per month

Prior to your ACL injury, did you consider martial arts to be your primary sport?

- ◯ Yes
- ◯ No

Which sport would you consider to be your primary sport?

____

Information about your ACL injury

(If multiple ACL injuries, please answer the following questions regarding your FIRST ACL injury that led to requiring surgery.)

When did you injure your ACL? (If multiple, please record the date of your initial injury.) __________________________________

Which side was your ACL injury?
- ◯ Left
- ◯ Right

How did you injure your ACL?
- ◯ Martial arts or combat sports
- ◯ Other

If other, please describe. (Include sport, contact vs. non-contact) ____

Did your injury occur while sparring with an opponent?
- ◯ Yes
- ◯ No

Which fighting maneuver caused your injury?
- ◯ I was taken down
- ◯ I was taking my opponent down
- ◯ I was passing guard
- ◯ I got my guard passed
- ◯ I was attempting a submission
- ◯ My opponent was attempting a submission
- ◯ I was sweeping my opponent
- ◯ I was being swept
- ◯ I don't know
- ◯ Other

If other, describe what fighting maneuver caused your injury. __________________________________

Was the injury during warm-up, drilling techniques, sparring or during competition?
- ◯ Warm-up
- ◯ Drilling technique
- ◯ Sparring
- ◯ Competition

Comments ____

Information about your ACL reconstruction

(If multiple, please answer these questions in relation to your first ACL reconstruction.)

What year was your ACL reconstruction done in? (If multiple, please record the year of your first ACL surgery.) __________________________________

Have you had any orthopaedic procedures on your lower extremities other than your ACLR?
- ◯ Yes
- ◯ No

How many procedures have you had on your lower extremities other than your ACL reconstruction due to the martial arts injury?	◯ 1 ◯ 2 ◯ 3 ◯ 4 ◯ 5
Please List the Type of Procedure and the Year that it was performed<br><br>(Please clarify side. i.e., right knee replacement, left hip repair, etc...)	
______ Procedure Name______ Year______	
______ Procedure Name______ Year______	
______ Procedure Name______ Year______	
______ Procedure Name______ Year______	
______ Procedure Name______ Year______	
After your ACL reconstruction, how long did you receive physical therapy?	◯ None<br>◯ Less than 1 month<br>◯ 1-3 months<br>◯ 3-6 months<br>◯ 6-9 months<br>◯ 9-12 months<br>◯ Greater than 12 months
Did you have any complications after your ACL reconstruction such as infection, DVT, pneumonia, re-operation etc.?	◯ Yes<br>◯ No
If yes, please describe.	__________________________________
Did you have another ACL injury after your first ACL reconstruction?	◯ No<br>◯ Yes, same knee<br>◯ Yes, other knee<br>◯ Yes, both knees

Sports participation AFTER your ACL injury

(If multiple, please answer these questions in relation to recovery from your first ACL reconstruction.)

Did you have ANY other injuries in the same knee after your ACL reconstruction?	◯ Yes<br>◯ No
If yes, please specify.	__________________________________
Did you return to martial arts AFTER your ACL injury?	◯ Yes<br>◯ No

If not, what kept you from returning to training?

- ◯ Pain
- ◯ Lack of mobility
- ◯ Fear of getting reinjured
- ◯ Persisting instability
- ◯ Doctor's advice
- ◯ Other (not ACL injury related)

If other, please explain.

____

How long did it take you to return to light martial arts training? (Non-contact/no sparring, drills only.)

______

When were you cleared to fully return to martial arts training? (sparring/contact included)

______

When was your first martial arts competition after your ACL surgery?

- ◯ Within 3 months after surgery
- ◯ Within 6 months after surgery
- ◯ Within 9 months after surgery
- ◯ Within 12 months after surgery
- ◯ Within 1.5 years after surgery
- ◯ Within 2 years after surgery
- ◯ More than 2 years after surgery
- ◯ I never competed before surgery
- ◯ I never returned to competition level after surgery (due to my ACL injury)
- ◯ I never returned to competition level after surgery (non-ACL related reason)

Did you return to the same competitive level as before your ACL injury?

- ◯ Yes
- ◯ No
- ◯ Reached a higher level

Current sports participation

What is your current belt level?

____

How often do you currently participate in martial arts?

- ◯ 4 to 7 times per week
- ◯ 1 to 3 times per week
- ◯ 1 to 3 times per month
- ◯ Less than one time per month

Does your knee injury still affect your participation in martial arts?

- ◯ Not at all
- ◯ Mildly
- ◯ Moderately
- ◯ Severely
- ◯ Extremely

Return to work

Question	Response
Have you returned to normal work activity?	◯ Not applicable, I was not working prior to my injury<br>◯ No, I have not returned to work<br>◯ Yes, I have returned to work in a limited or modified role<br>◯ Yes, I have returned to work without restrictions
If you have not returned to your normal work activity OR only returned in a limited role, please indicate the reason(s) below:	☐ My physician/surgeon has not cleared me to participate in work activities.<br>☐ My knee injury does not allow me to participate in work activities because of ongoing knee problems.<br>☐ I am not confident in my knee.<br>☐ I am worried about re-injury.<br>☐ I am permanently ineligible to participate in work activities because of my knee injury (e.g., permanent medical disability)<br>☐ Other reason
Please briefly specify "other" reason for not returning to work or for returning in a limited role:	______________________________
Before your knee injury, what was your employment status?	◯ Work regular duty, full time<br>◯ Work regular duty, part time<br>◯ Work light duty or modified position full time<br>◯ Work light duty or modified position part time<br>◯ Homemaker (not working outside of the home)<br>◯ Student only (not currently working)<br>◯ Retired (not due to health status)<br>◯ Temporarily unable to work due to health status<br>◯ Permanently unable to work or retired due to health status<br>◯ Unemployed - out of work for less than 1 year<br>◯ Unemployed - out of work for 1 year or more
What type of work did you do prior to your injury?	◯ Very heavy - Lift, on an occasional basis, over 100 pounds with frequent or constant lifting in excess of 50 pounds.<br>◯ Heavy - Lift, on an occasional basis, a maximum of 100 pounds, with more frequent or constant lifting of 50 pounds<br>◯ Moderately Heavy - Lift, on an occasional basis, a maximum of 80 pounds, with more frequent or constant lifting of 40 pounds<br>◯ Medium - Lift, on an occasional basis, a maximum of 50 pounds, with more frequent or constant lifting of 25 pounds<br>◯ Light - Lift, on an occasional basis, a maximum of 20 pounds with more frequent or constant lifting of 10 pounds

Currently, what is your employment status?	◯ Work regular duty, full time<br>◯ Work regular duty, part time<br>◯ Work light duty or modified position full time<br>◯ Work light duty or modified position part time<br>◯ Homemaker (not working outside of the home)<br>◯ Student only (not currently working)<br>◯ Retired (not due to health status)<br>◯ Temporarily unable to work due to health status<br>◯ Permanently unable to work or retired due to health status<br>◯ Unemployed - out of work for less than 1 year<br>◯ Unemployed - out of work for 1 year or more
What type of work are you currently doing?	◯ Very heavy - Lift, on an occasional basis, over 100 pounds with frequent or constant lifting in excess of 50 pounds.<br>◯ Heavy - Lift, on an occasional basis, a maximum of 100 pounds, with more frequent or constant lifting of 50 pounds<br>◯ Moderately Heavy - Lift, on an occasional basis, a maximum of 80 pounds, with more frequent or constant lifting of 40 pounds<br>◯ Medium - Lift, on an occasional basis, a maximum of 50 pounds, with more frequent or constant lifting of 25 pounds<br>◯ Light - Lift, on an occasional basis, a maximum of 20 pounds with more frequent or constant lifting of 10 pounds

Please indicate below the HIGHEST level of activity that you participated in BEFORE YOUR INJURY and the highest level you are able to participate in CURRENTLY.

Level 10 Competitive sports- soccer, football, rugby (national elite)

Level 9 Competitive sports- soccer, football, rugby (lower divisions), ice hockey, wrestling, gymnastics, basketball

Level 8 Competitive sports- racquetball or bandy, squash or badminton, track and field athletics (jumping, etc.), down-hill skiing

Level 7 Competitive sports- tennis, running, motorears speedway, handball

Recreational sports- soccer, football, rugby, bandy, ice hockey, basketball, squash, racquetball, running

Level 6 Recreational sports- tennis and badminton, handball, racquetball, down-hill skiing, jogging at least 5 times per week

Level 5 Work- heavy labor (construction, etc.)

Competitive sports- cycling, cross-country skiing

Recreational sports- jogging on uneven ground at least twice weekly

Level 4 Work- moderately heavy labor (eg. truck driving, etc.)

Level 3 Work- light labor (nursing, etc.)

Level 2 Work- light labor

Walking on uneven ground possible, but impossible to back pack or hike

Level 1 Work- sedentary (secretarial, etc.)

Level 0 Sick leave or disability pension because of knee problems

BEFORE INJURY level 0 ______ 10

(Place a mark on the scale above)

CURRENT level 0 ______ 10

(Place a mark on the scale above)
